# Stroke Diagnosis and Prediction Tool Using ChatGLM: Development and Validation Study

**DOI:** 10.2196/67010

**Published:** 2025-02-26

**Authors:** Xiaowei Song, Jiayi Wang, Feifei He, Wei Yin, Weizhi Ma, Jian Wu

**Affiliations:** 1 Department of Neurology Beijing Tsinghua Changgung Hospital, School of Clinical Medicine, Tsinghua University Beijing China; 2 Harbin Institute of Technology Harbin China; 3 Department of Neurology Beijing Geriatric Hospital Beijing China; 4 School of Biomedical Engineeering Tsinghua Medicine Tsinghua University Beijing China; 5 Institute for AI Industry Research Tsinghua University Beijing China

**Keywords:** stroke, diagnosis, large language model, ChatGLM, generative language model, primary care, acute stroke, prediction tool, stroke detection, treatment, electronic health records, noncontrast computed tomography

## Abstract

**Background:**

Stroke is a globally prevalent disease that imposes a significant burden on health care systems and national economies. Accurate and rapid stroke diagnosis can substantially increase reperfusion rates, mitigate disability, and reduce mortality. However, there are considerable discrepancies in the diagnosis and treatment of acute stroke.

**Objective:**

The aim of this study is to develop and validate a stroke diagnosis and prediction tool using ChatGLM-6B, which uses free-text information from electronic health records in conjunction with noncontrast computed tomography (NCCT) reports to enhance stroke detection and treatment.

**Methods:**

A large language model (LLM) using ChatGLM-6B was proposed to facilitate stroke diagnosis by identifying optimal input combinations, using external tools, and applying instruction tuning and low-rank adaptation (LoRA) techniques. A dataset containing details of 1885 patients with and those without stroke from 2016 to 2024 was used for training and internal validation; another 335 patients from two hospitals were used as an external test set, including 230 patients from the training hospital but admitted at different periods, and 105 patients from another hospital.

**Results:**

The LLM, which is based on clinical notes and NCCT, demonstrates exceptionally high accuracy in stroke diagnosis, achieving 99% in the internal validation dataset and 95.5% and 79.1% in two external test cohorts. It effectively distinguishes between ischemia and hemorrhage, with an accuracy of 100% in the validation dataset and 99.1% and 97.1% in the other test cohorts. In addition, it identifies large vessel occlusions (LVO) with an accuracy of 80% in the validation dataset and 88.6% and 83.3% in the other test cohorts. Furthermore, it screens patients eligible for intravenous thrombolysis (IVT) with an accuracy of 89.4% in the validation dataset and 60% and 80% in the other test cohorts.

**Conclusions:**

We developed an LLM that leverages clinical text and NCCT to identify strokes and guide recanalization therapy. While our results necessitate validation through widespread deployment, they hold the potential to enhance stroke identification and reduce reperfusion time.

## Introduction

### Background

Stroke ranks among the leading causes of morbidity and mortality worldwide. The overall burden of stroke, in terms of the absolute number of individuals affected or left disabled by the condition, has been increasing, particularly in China [1-3]. There are significant disparities in stroke incidence, prevalence, acute care, rehabilitation, risk factor management, and outcomes [4]. In recent years, substantial progress has been made in stroke diagnosis and treatment, with effective recanalization therapies for acute ischemic stroke established to significantly improve clinical prognosis [5,6]. However, treatment for acute ischemic stroke is highly time-dependent. Despite ample evidence supporting intravenous thrombolysis (IVT) and endovascular therapy for acute ischemic stroke, the number of patients who arrive at hospitals and are eligible for IVT remains limited, only 10% in the United States and 6% in China, with the global rate for IVT being lower than 5%. In addition, fewer than 100,000 thrombectomies are performed annually, despite the high incidence of ischemic strokes caused by large vessel occlusion (LVO). Access to recanalization therapies also varies across different regions [7,8]. Furthermore, the limited number of stroke neurologists is insufficient to meet the growing number of new stroke cases each year. Consequently, many patients in underdeveloped areas do not receive adequate care in the event of a sudden stroke. Inequities in stroke care are prevalent, and the costs also vary significantly [4,9-11].

The emergence of artificial intelligence (AI) and its application in health care has shown great promise in bridging existing gaps in medical practice [12,13]. Several studies have demonstrated the potential of large language models (LLMs) in managing various diseases [14,15]. In addition, LLMs have proven their effectiveness in medical diagnostics. For example, the Med-PaLM model illustrates the capabilities of LLMs in enhancing medical decision-making, achieving performance levels comparable to those required for US medical licensing examinations [16]. Furthermore, LLMs are used to extract summaries from electronic health records, making medical data more accessible and manageable for health care providers [17]. The integration of AI in clinical decision-making could reduce interrater variability in routine practice and facilitate the extraction of critical information, thereby improving the identification of patients with stroke, predicting treatment responses, and enhancing patient outcomes [14].

### Objective

The objective of this study was to develop an acute stroke diagnosis tool that guides key therapies based on ChatGLM-6B and to verify its accuracy in diagnosing and predicting strokes. The application of this model in primary care could enhance the standard workflow for stroke diagnosis, identify patients who could benefit from recanalization, and facilitate risk prediction.

## Methods

### Study Design

We used a retrospective cohort for model training and internal validation while using a prospective cohort from various hospitals for external validation.

### Ethical Considerations

The study was conducted in accordance with the Declaration of Helsinki, and the protocol was approved by the Beijing Tsinghua Changgung Hospital Ethics Committee (23641-0-01). Written informed consent was waived due to the analysis of electronic health records, and all research data used in this study are anonymous.

### Data Acquisition

Training data were obtained from Beijing Tsinghua Changgung Hospital, a comprehensive stroke center and teaching hospital. Subjects admitted to the emergency room (ER) between January 2016 and January 2024 were screened continuously. Of the 341,026 patients admitted to the study hospital’s ER during this period, 33,762 presented with focal neurological symptoms and were referred to the neurology ER. Among these, 12,600 were diagnosed with cerebrovascular disease (*ICD-10* [*International Statistical Classification of Diseases and Related Health Problems* 10th Revision]), and 991 were diagnosed with stroke and exhibited symptoms within 24 hours. The control group was randomly selected from patients admitted to the neurology ER who were diagnosed with noncerebrovascular diseases, ensuring a match for age, sex, admission time, and attending physicians after removing duplicate admissions. A focal neurologic deficit refers to a dysfunction in the nerves, spinal cord, or brain that affects a specific area of the body, such as the left side of the face, the right arm, or even a localized region like the tongue. In addition, issues related to speech, vision, and cognitive function are also classified as focal neurological deficits; the number of cases in each symptom category was shown in Multimedia Appendix 1. A total of 1885 cases satisfying the criteria above were included to form the training set. An additional group of patients from the same hospital (n=230) and another hospital (n=105) over the course of another month was used for the external test.

**Figure 1:**
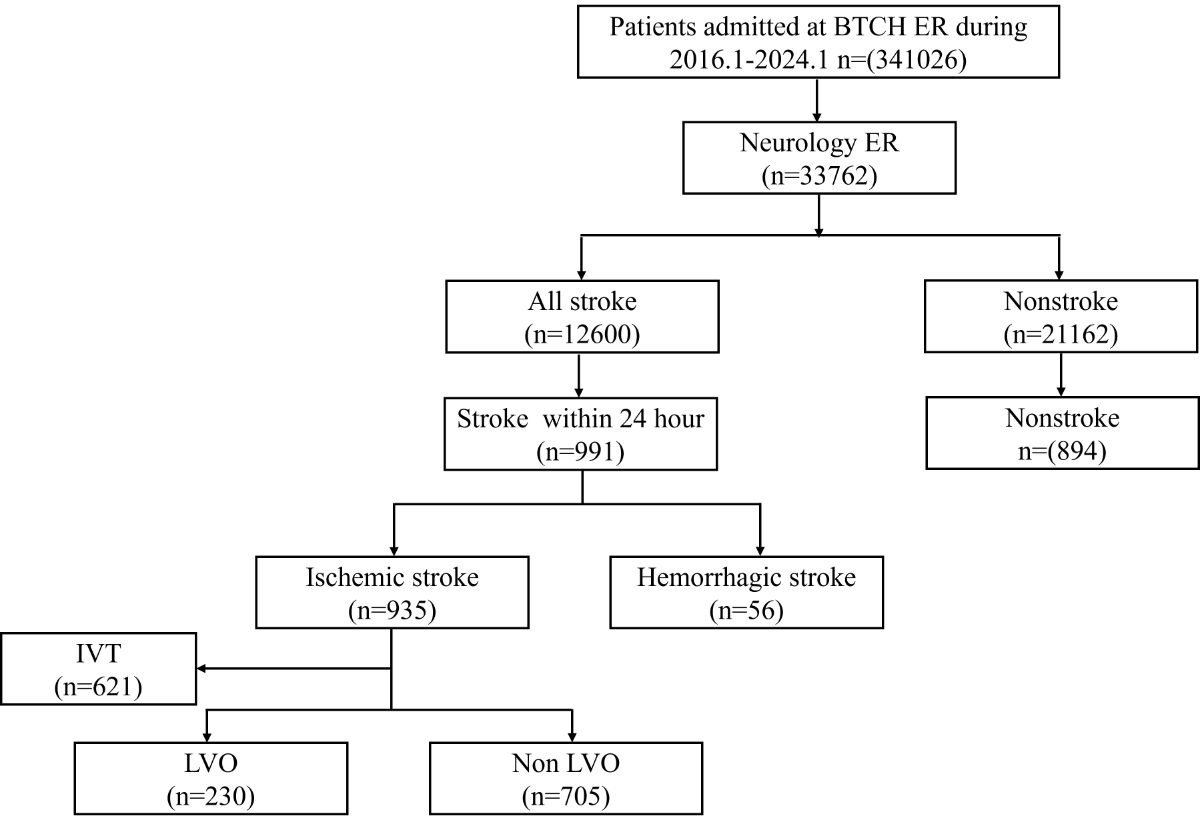
Flowchart of training dataset inclusion. BTCH: Bingjing Tsinghua Changgung Hospital; ER: emergency room; IVT: intravenous thrombolysis; LVO: large vessel occlusion.

Demographic characteristics, emergency clinical notes, radiological images and reports, as well as other relevant examination records, were obtained from the hospital information system. The subjects were categorized into stroke and nonstroke groups based on the discharge diagnosis. Among the patients with stroke, further classification was adopted to divide them into ischemic and hemorrhagic strokes. Patients with ischemic stroke who received IVT or had LVO, confirmed by computed tomography angiography or magnetic resonance angiography, were also identified, regardless of whether they underwent endovascular therapy. In this study, large vessel occlusion is defined as ischemia resulting from the obstruction of a corresponding large vessel, which includes the anterior cerebral artery, middle cerebral artery, posterior cerebral artery, basilar artery, and vertebral artery. A detailed description of the subject screening process is provided in Figure 1. All stroke diagnoses and workflows adhered to the recommendations outlined in the acute ischemic stroke management guidelines [18].

### Data Preprocessing

Each record included demographic details such as age, sex, mode of arrival to the hospital (via emergency medical dispatch system or otherwise), main complaints, present medical history related to the current event, previous medical history, neurological examination, auxiliary examinations (including blood tests, electrocardiogram, and brain computed tomography as radiology reports), allergy history, marital and reproductive history, personal history, and family history. Notably, some records contained missing items for specific clinical parameters. We addressed these gaps by indicating “information not provided.”

It was observed that some records included details such as past medical history and allergy history within the section on the history of the current illness. These details were extracted using methods such as regular expressions. In addition, information related to COVID-19 epidemiological investigations was present; however, since it was irrelevant to the diagnosis of the patient’s condition, it was removed. All cases were randomly divided into training and internal validation datasets in a 9:1 ratio.

### Foundation Model: ChatGLM-6B

To address the aforementioned challenges, we used ChatGLM-6B as the foundational model, which serves as the core of our acute stroke diagnosis tool, enabling high-precision analysis of clinical data. The ChatGLM-6B model was chosen due to its relatively low deployment requirements for hospital environments and its outstanding performance capabilities.

### Prompt

**Figure 2:**
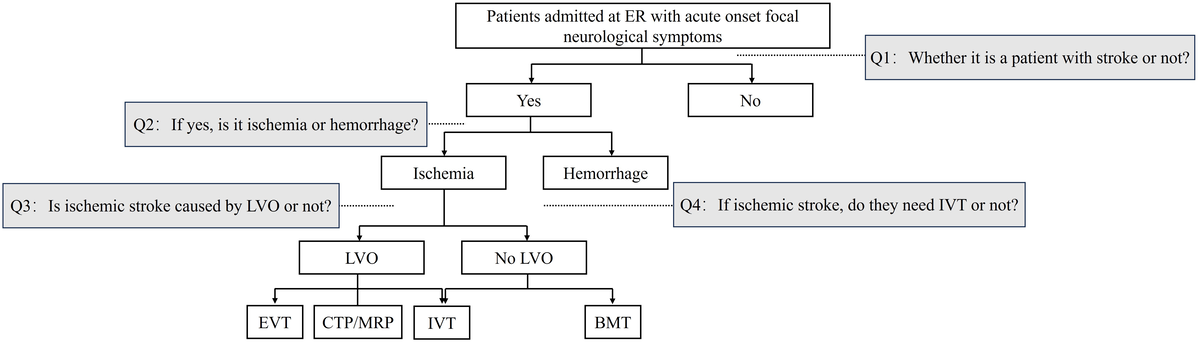
Acute ischemic stroke management workflow and key issues. BMT: best medical treatment; CTP: computed tomography perfusion; ER: emergency room; EVT: endovascular thrombectomy; IVT: intravenous thrombolysis; LVO: large vessel occlusion; MRP: magnetic resonance perfusion.

**Figure 3:**
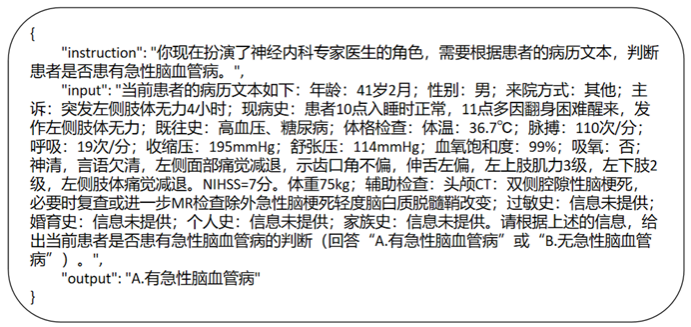
An example of instruction fine-tuning format.

The following prompt was used:

“As a neurology physician, please review the clinical notes and provide a final diagnosis based on the information provided. Kindly address the following questions: (1) Is the patient experiencing a stroke? (2) If so, is it ischemic or hemorrhagic? (3) If it is an ischemic stroke, does the patient require IVT? (4) If it is an ischemic stroke, is it caused by large vessel occlusion?” (see Figure 2 and Figure 3).

For clarity, when responding to the second, third, and fourth questions, we extracted specific subsets from the original dataset, ensuring that each question presented only two options. For example, when addressing the second question, we exclusively extracted records of stroke cases from the training, validation, and test sets.

### Features of the Medical Records

The electronic medical records contain a wealth of information, including primary complaints, medical history related to the current event, previous medical history, and more. Based on the completeness of various entries, certain combinations of entries are manually configured to select the optimal combination for different tasks, as illustrated in the textbox below (Textbox 1).

**Different electronic health record entry combinations.**

**Electronic health record entry combinations**

- （ALL) Age, sex, mode of arrival, main complaints, medical history for this event, previous medical history, neurological examination, auxiliary examination, allergy history, marital and reproductive history, personal history, and family history.
- Age, sex, mode of arrival, main complaints, medical history for this event, previous medical history, neurological examination, auxiliary examination, allergy history, marital and reproductive history.
- Age, sex, mode of arrival, main complaints, medical history for this event, previous medical history, neurological examination, and auxiliary examination.
- Age, sex, mode of arrival, main complaints, medical history for this event, neurological examination, and auxiliary examination.
- Age, sex, mode of arrival, medical history for this event, previous medical history, neurological examination, auxiliary examination.
- Age, sex, mode of arrival, medical history for this event, neurological examination, auxiliary examination.
- Age, sex, medical history for this event, previous medical history, neurological examination, and auxiliary examination.
- Age, sex, medical history for this event, neurological examination, and auxiliary examination.

### In-Context Learning

In-context learning is a key ability of LLMs that allows them to handle new tasks with just a few examples. Few-shot learning is considered one of the most effective methods for in-context learning [19]. By using prompting techniques, foundational models can quickly adapt to a specific domain and learn to follow the task format with just a few demonstrations. This approach uses a mechanism to identify examples based on their similarity to the current case [20].

To identify representative few-shot examples, we followed this procedure: for each test example, we selected k training examples that are semantically similar using k-nearest neighbors clustering within the embedding space. Specifically, we used ChatGLM-6B to embed both training and test questions into vector representations. Then, for each test question (x), we retrieved its k-nearest neighbors from the training set. Unlike fine-tuning, dynamic few-shot learning uses the training data without requiring extensive updates to the model parameters.

### Instruction Fine-Tuning and LoRA

In this study, we used instruction tuning and low-rank adaptation (LoRA) techniques to enhance the performance of LLMs on specific medical record tasks [21,22]. Instruction tuning is a training method that provides explicit task instructions to the model during the training process, enabling it to better understand and execute these instructions, thereby improving its performance on targeted tasks. Conversely, LoRA technology adapts the model’s weight matrices through low-rank modifications, allowing for fine-tuning of model behaviors without the need to retrain the entire model. This approach improves both training and inference efficiency while preserving the model’s complexity.

Specifically, we first developed distinct task instructions based on the four identified issues, which represent critical questions in the stroke diagnosis and treatment workflow (Figure 2). These instructions formed the instruction component of the directive. The primary entries from the medical records were concatenated to create the medical record text, which served as the Input component of the instruction. Finally, the labels corresponding to the questions were designated as the Output component of the instruction. To select the primary features from the medical records, combinations of features were manually chosen based on the completeness of various characteristics within the records (Textbox 1). The best-performing features under a zero-shot scenario, without fine-tuning, were then selected for use during the fine-tuning process. An example is provided below.

### Tool Chaining with LLMs

Tool chaining with LLMs involves the integration of various pretrained tools, such as vision models, to enhance the capabilities of LLMs. This process does not necessitate additional model training. For instance, in implementations like Visual ChatGPT and X-GPT, an LLM can assign specific tasks to the appropriate tools to manage subtasks that exceed its inherent capabilities [23,24].

In this study, we provided noncontrast computed tomography scans along with the corresponding radiology reports. By using a toolchain approach, we integrated these examination results into the medical records text and input them collectively into the large model, thereby eliminating the need for any additional tools. Furthermore, we compared the model’s performance with and without tool chaining, selecting the more effective option for various tasks.

### Parameter

The parameter settings for LoRA fine-tuning are as follows: LoRA_rank is set to 8, batch size to 6, gradient accumulation steps to 1, max steps to 2000, fp16 is enabled, and the learning rate is set at 5e-5.

## Results

### Overview

A total of 1885 cases were included in the training set, comprising 991 stroke cases and 894 nonstroke cases. Among the stroke cases, there were 935 instances of ischemic stroke; of these, 621 patients received IVT, and 230 presented with large vessel occlusion, which was confirmed by magnetic resonance angiography or computed tomography angiography.

### Performance on the Validation Set

We use the performance of the LLM in zero-shot and few-shot (3-shot) settings as the baseline, while the results obtained from fine-tuning serve as the final outcomes.

For zero-shot and few-shot scenarios, we experimented with various combinations of entries and the use of tools, ultimately selecting the most effective approach. For fine-tuning, we adopted the best method identified in the zero-shot scenario for adjustments and evaluated it under this framework. The primary results are presented in Table 1.

**Table 1:** Performance of large language models on stroke diagnosis on internal validation set using different methods.

Question and method			Approach		Accuracy		Sensitivity		Specificity
**Whether it is a patient** **with** **stroke or not?**									
	Zero-shot	No.1^a^+Tool^b^		0.713		0.702		0.722	
	Few-shot (3)	No.2+Tool		0.784		0.837		0.722	
	Fine-tune	No.1+Tool		0.99		1		0.978	
**If yes, is it ischemia or hemorrhage?**									
	Zero-shot	No.1+Tool		0.606		0.564		1	
	Few-shot (3)	No.8		0.894		0.883		1	
	Fine-tune	No.1+Tool		1		1		1	
**If ischemic stroke, do they need intravenous thrombosis or not?**									
	Zero-shot	No.8+Tool		0.617		0.776		0.222	
	Few-shot (3)	No.3		0.649		0.776		0.333	
	Fine-tune	No.8+Tool		0.894		0.985		0.667	
**Is ischemic stroke caused by LVO or not?**									
	Zero-shot	No.8		0.585		0.625		0.571	
	Few-shot (3)	No.4+Tool		0.713		0.417		0.814	
	Fine-tune	No.8		0.8		0.5		0.9	

^a^No.x: the xth entry combination shown in Textbox 1.

^b^Tool: using the tool chaining, ie, adding imaging examination results.

It can be observed that after few-shot learning, the model’s performance significantly improved on questions 1, 2, and 4, with some improvement also noted for question 3. Following the application of LoRA, there was a substantial enhancement in performance across all questions.

### Overall Performance Across Different Datasets

Using the fine-tuned weights, the same tests were conducted on the test set. Specifically, because the electronic medical records provided by other hospitals lacked information on the mode of arrival, marital status, and reproductive history, as well as imaging examination results, these components were excluded from the input. Instead, the “nervous system” data, which was available in this dataset but not originally included, was incorporated. The main results are presented in Table 2. For the key procedures in the stroke diagnosis workflow, the model demonstrates relatively satisfactory performance in stroke diagnosis, classification, and prediction.

**Figure 4:**
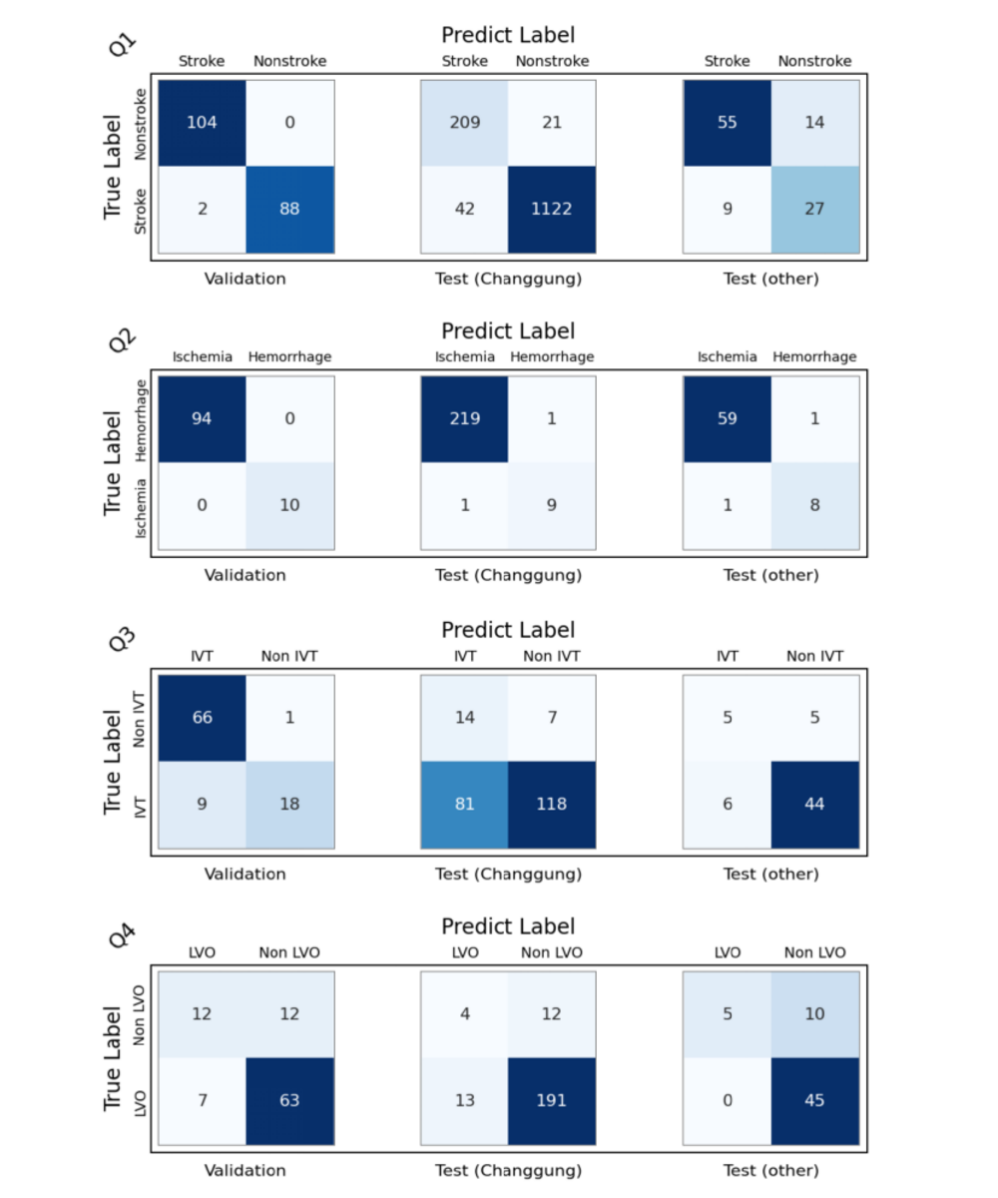
Confusion matrix of large language models on stroke diagnosis workflow key issues. IVT: intravenous thrombolysis; LVO: large vessel occlusions.

The model demonstrates exceptionally high accuracy in stroke diagnosis, achieving 99% in the internal validation cohort. Further validation across different cohorts and other stroke centers indicates that the accuracy remains at 95.5% within the same hospital and 79.1% in other hospitals. This model exhibits excellent performance in identifying both ischemic and hemorrhagic strokes, with accuracy reaching as high as 100% in internal validation and 99.1% and 97.1% in external validation. Regarding IVT for ischemic stroke, the model’s accuracy ranges from 60% to 89.4%. In addition, the model is capable of identifying large vessel occlusions, with prediction accuracy reaching up to 80% across different cohorts. However, it is important to note that while the model demonstrates high specificity, it has limited sensitivity (see Table 2 and Figure 4).

**Table 2:** Performance of large language models on stroke diagnosis based on low-rank adaptation on both internal validation and external test set.

Question and dataset						Accuracy		Sensitivity		Specificity	
**Whether it is a patient with stroke or not?**											
	**Validation**				0.99		1		0.978		
	**Test**										
			Changgung (n=230)		0.955		0.909			0.964	
			Other (n=105)		0.791		0.812			0.75	
**If yes, is it ischemia or hemorrhage?**											
	**Validation**				1		1		1		
	**Test**										
			Changgung (n=230)		0.991		0.996			0.9	
			Other (n=105)		0.971		0.983			0.889	
**If ischemic stroke, do they need intravenous thrombosis or not?**											
	**Validation**				0.894		0.985		0.667		
	**Test**										
			Changgung (n=230)		0.6		0.667			0.593	
			Other (n=105)		0.817		0.5			0.88	
**If ischemic stroke, is it caused by LVO or not?**											
		**Validation**				0.8		0.5		0.9	
	**Test**										
			Changgung (n=230)		0.886		0.25			0.936	
			Other (n=105)		0.833		0.333			1	

We also conducted zero-shot and few-shot tests on the test set, using the optimal combinations selected from the validation set for both scenarios. The results were compared with those obtained from LoRA, as shown in Table S3 in Multimedia Appendix 2 and Table S4 in Multimedia Appendix 3.

### Verify the Effectiveness of Selecting the Optimal Entry Combinations

Since fine-tuning with various entry combinations is time-consuming, we focus on fine-tuning based on the best combination identified from the zero-shot results. The optimal combinations for questions 1 and 2 consist of all entries combined with medical imaging examination results. We then compare the best combinations for questions 3 and 4 against the all-inclusive combination of entries and medical imaging examination results on the validation set. This comparison aims to demonstrate the effectiveness of selecting specific combinations rather than simply opting for the maximum number of entries (see Table 3). For question 3, the optimal combination yielded significantly better performance than using all available information. In question 4, while the accuracy of the optimal combination was slightly lower than that of the all-inclusive approach, it exhibited a more balanced performance in terms of sensitivity and specificity; the *P* values for the comparison between best and all among questions 3 and 4 were <0.001 and 0.017, respectively.

**Table 3:** Comparison between selecting the optimal combination and selecting all information on the validation set.

Question and method^a^		Accuracy	Sensitivity	Specificity
**If ischemic stroke, do they need intravenous thrombosis or not?**				
	Best^a^	0.8936	0.9851	0.6667
	All^b^	0.8404	0.9701	0.5185
**If ischemic stroke, is it caused by LVO^c^ or not?**				
	Best	0.8	0.5	0.9
	All	0.8085	0.3333	0.9714

^a^Best: the best entry combinations for questions.

^b^All: all entries and medical imaging examination results.

^c^LVO: large vessel occlusions.

## Discussion

### Principal Findings

To the best of our knowledge, this is the first LLM developed for stroke diagnosis. Domain-specific models are typically more efficient than general-purpose models. Our model has demonstrated excellent performance in stroke diagnosis and classification. Compared to other LLMs, ChatGLM-6B has an affordable advantage in health care purposes, which is critically important in the medical domain. The easy deployment of our model could undoubtedly facilitate the wide-scale application in primary care.

### A Review of Previous Stroke Diagnosis Tool and Their Limitations

Addressing inequities in acute stroke care necessitates a thorough examination of each component of regional workflow, particularly the collaboration among various stroke centers [19]. In addition to raising awareness of stroke symptoms among high-risk populations, the most critical step is to identify patients with stroke as early as possible at primary stroke centers. Although several scales existed for stroke diagnosis, they often exhibited limited sensitivity and specificity. The Cincinnati Prehospital Stroke Scale (CPSS) is a clinical evaluation tool for stroke diagnosis, comprising three items: facial droop, upper limb weakness, and speech difficulties. If a patient presents with one of these three symptoms, there is a 72% likelihood that they are experiencing a stroke; if all three symptoms are present, the probability increases to 85% [20,21]. The 3-Item Stroke Scale (3-ISS) is used to identify proximal vessel occlusion (specifically T-segment or M1-segment occlusion of the middle cerebral artery), with an overall accuracy of 86%, sensitivity of 67%, and specificity of 92% [22]. However, these evaluations are primarily based on clinical symptoms, which can lead to an underestimation of stroke diagnoses in atypical patients and an overestimation in individuals with other neurological conditions. In addition, there are several large vessel occlusion scales based on imaging techniques, such as the Alberta Stroke Program Early CT Score (ASPECTS) and multimodal perfusion imaging. Computed tomography perfusion is the most widely used multimodal imaging method in comprehensive stroke centers, and numerous automated postprocessing workstations, such as Rapid AI and Viz.AI, assist in LVO and penumbra calculations. These technologies can enhance LVO workflow to some extent and reduce door-to-groin time [12,23]. However, there are limitations to this assessment. First, computed tomography perfusion may not be available in some primary care settings. Second, automated postprocessing workstations are not universally implemented across all hospitals. Third, the varying algorithms used by different products can also impact the final diagnosis [24].

### Clinical Implications

Our results demonstrated the significant potential of LLMs in stroke diagnosis and management, particularly in differentiating stroke mimics. The relatively low accuracy of stroke diagnosis in external validation may be attributed to discrepancies in the clinical notes template and deficiencies in radiology reports from other hospitals. Our model, which is based on initial visit records and brain computed tomography scans, achieved an accuracy of nearly 80% in identifying LVO and recommending IVT. This level of accuracy is comparable to that of an experienced stroke neurologist and surpasses the performance of the Alberta Stroke Program Early CT Score [25]. Previous studies also support the notion that multimodal LLMs, when used to evaluate the prognosis of hemorrhagic stroke, can significantly enhance predictive performance compared to single modalities, such as clinical notes or imaging [26]. In contrast to other disease-specific language representation models, such as stroke-BERT [27], ChatGLM-6B is a general-purpose language model with extensive capabilities in language understanding and generation. This allows it to capture semantic and contextual information more comprehensively when processing diverse medical records. In addition, the GLM framework supports multimodal data input and can integrate information from images, which is particularly crucial in the diagnosis of cerebrovascular diseases. When compared to traditional machine learning and deep learning models, LLMs often demonstrate superior performance across a range of language-based tasks. For instance, LLMs have been successfully applied in diagnosing medical conditions, assisting in medical education, and even generating medical documentation, where they can outperform more traditional models that require extensive feature engineering and fine-tuning for each specific task [17]. This is the first LLM tool in stroke diagnosis; the model can give some guidance on stroke diagnosis, classification, and management recommendations based on the primary information. As we know, there exist great discrepancies in stroke care across different hospitals. Stroke centers were more likely to receive thrombolytic therapy and had lower 30-day mortality rates [28]. Thus, the deployment of this model in primary care, especially where stroke neurologists are in high demand, could not only improve the ability of stroke diagnosis early and accurately, but also improve reperfusion therapy for patients who are very likely to have LVO, and they can be transferred to advanced stroke center as soon as possible, saving both time and economic costs. A previous study indicated that the use of artificial intelligence decision support in a hyperacute stroke pathway facilitates decision-making and can improve the rate and time of reperfusion therapies in a hub-and-spoke system of care [29]. There are also data supports that transfer-in patients had similar in-hospital mortality rates compared with front-door patients [30].

### Limitations and Future Work

This study has several limitations. First, it only included data from two centers in the real world for external validation, and the data were exclusively from the Chinese population, which could introduce some data bias. We still need more data from different stroke centers to validate the accuracy of our model. Second, electronic health records may be fragmented and the clinical notes could also be subjective and depend on different physicians, which could possibly have an effect on the test results. However, the advanced language comprehension capabilities of LLMs enable them to extract crucial information from diverse and complex clinical notes, even when the documentation varies in style and contains incomplete data. This capacity significantly enhances the quality and efficiency of medical diagnostics. Finally, despite the satisfactory performance of this model in stroke diagnosis, as an LLM, the hallucination is unavoidable. We strive to minimize the possibility by adjusting the temperature, paying more attention to the prompt project, and verifying the results in an external dataset. As an auxiliary diagnostic tool, we must interpret its suggestions with caution.

### Conclusion

In this study, we developed an LLM for stroke diagnosis with quite good performance in in-context understanding and reasoning skills by selecting optimal entry combinations, using tools, and fine-tuning with LoRA, proving its effectiveness. Our model also performs well in handling missing values, whether individual data points lack information on certain entries or the dataset itself lacks corresponding entries. In general, AI holds promise as a tool to ease the stroke neurologist’s shortage, improve daily workflow, and supply unique diagnostic insights by analyzing data simultaneously from several sources, including neurological history and examination, blood tests, and images.

## Appendix

Multimedia Appendix 1 Symptom categories and number of cases in training set.

Multimedia Appendix 2 Performance of LLMs on stroke diagnosis on test set (changgung).

Multimedia Appendix 3 Performance of LLMs on stroke diagnosis on test set(other).

## Abbreviations

3-ISS: 3-Item Stroke Scale
AI: artificial intelligence
ASPECTS: Alberta Stroke Program Early CT Score
CPSS: Cincinnati Prehospital Stroke Scale
ER: emergency room
ICD-10: International Statistical Classification of Diseases and Related Health Problems 10th Revision
IVT: intravenous thrombolysis
LLM: large language model
LoRA: low-rank adaptation
LVO: large vessel occlusion
