# Supplementary figures and images for: Stroke Diagnosis and Prediction Tool Using ChatGLM: Development and Validation Study
Source: J Med Internet Res. 2025 Feb 26;27:e67010. doi: 10.2196/67010 (PMC11904371; doi:10.2196/67010)

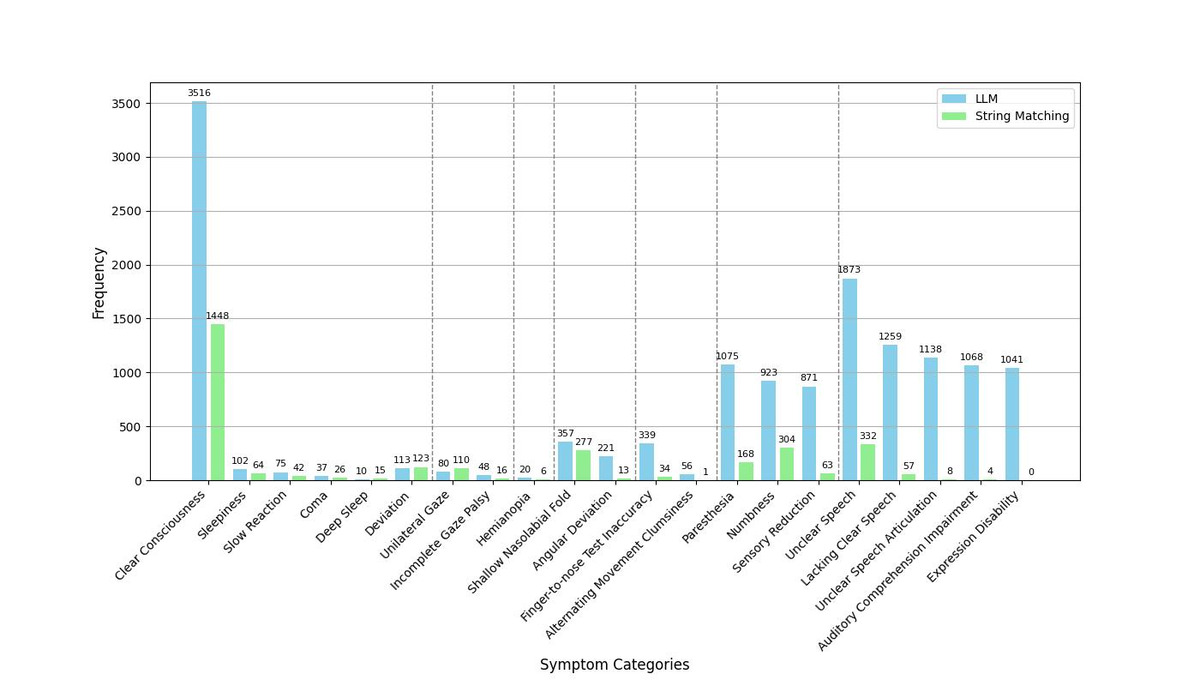

Supplement: Multimedia Appendix 1 [file jmir_v27i1e67010_app1.png]
