# Supplementary material for: Stroke Diagnosis and Prediction Tool Using ChatGLM: Development and Validation Study
Source: J Med Internet Res. 2025 Feb 26;27:e67010. doi: 10.2196/67010 (PMC11904371; doi:10.2196/67010)
Supplement: Multimedia Appendix 2 [file jmir_v27i1e67010_app2.docx]

**Table 4 Performance of LLMs on stroke diagnosis on test set (changgung)**

| **Question** | **Method** | **Accuracy** | **Sensitivity** | **Specificity** |
| --- | --- | --- | --- | --- |
| **Whether it is a patient with stroke or not?** | Zero-shot | 0.601 | 0.713 | 0.579 |
|  | Few-shot(3) | 0.720 | 0.774 | 0.709 |
|  | Fine-tune | **0.955** | **0.909** | **0.964** |
| **If yes, is it ischemia or hemorrhage?** | Zero-shot | 0.417 | 0.391 | **1.000** |
|  | Few-shot(3) | 0.896 | 0.900 | 0.800 |
|  | Fine-tune | **0.991** | **0.996** | 0.9 |
| **If ischemic stroke, do they need intravenous thrombosis or not?** | Zero-shot | 0.314 | **0.714** | 0.273 |
|  | Few-shot(3) | 0.300 | **0.714** | 0.256 |
|  | Fine-tune | **0.600** | 0.667 | **0.593** |
| **If ischemic stroke, is it caused by LVO or not?** | Zero-shot | 0.427 | 0.500 | 0.492 |
|  | Few-shot(3) | 0.696 | **0.563** | 0.706 |
|  | Fine-tune | **0.886** | 0.25 | **0.936** |
