# Supplementary material for: Stroke Diagnosis and Prediction Tool Using ChatGLM: Development and Validation Study
Source: J Med Internet Res. 2025 Feb 26;27:e67010. doi: 10.2196/67010 (PMC11904371; doi:10.2196/67010)
Supplement: Multimedia Appendix 3 [file jmir_v27i1e67010_app3.docx]

**Multimedia Appendix 3**

**Table 5 Performance of LLMs on stroke diagnosis on test set(other)**

| **Question** | **Method** | **Accuracy** | **Sensitivity** | **Specificity** |
| --- | --- | --- | --- | --- |
| **Whether it is a patient with stroke or not?** | Zero-shot | 0.629 | 0.754 | 0.389 |
|  | Few-shot(3) | 0.724 | **1.000** | 0.195 |
|  | Fine-tune | **0.791** | 0.812 | **0.750** |
| **If yes, is it ischemia or hemorrhage?** | Zero-shot | 0.768 | 0.733 | **1.000** |
|  | Few-shot(3) | **0.971** | 0.9667 | **1.000** |
|  | Fine-tune | **0.971** | **0.983** | 0.889 |
| **If ischemic stroke, do they need intravenous thrombosis or not?** | Zero-shot | 0.300 | **1.000** | 0.163 |
|  | Few-shot(3) | 0.367 | 0.700 | 0.300 |
|  | Fine-tune | **0.817** | 0.500 | **0.880** |
| **If ischemic stroke, is it caused by LVO or not?** | Zero-shot | 0.267 | **0.928** | 0.071 |
|  | Few-shot(3) | 0.500 | 0.667 | 0.444 |
|  | Fine-tune | **0.817** | 0.333 | **1.000** |
